# Development and clinimetric assessment of a performance-based functional vision tool in visually impaired children

**DOI:** 10.3389/fped.2023.1275726

**Published:** 2023-11-01

**Authors:** Fatemeh Ghasemi Fard, Hooshang Mirzaei, Seyed Ali Hosseini, Abbas Riazi, Abbas Ebadi, Narges Hooshmandzadeh

**Affiliations:** ^1^ Pediatric Neurorehabilitation Research Center, University of Social Welfare and Rehabilitation Sciences, Tehran, Iran; ^2^ Department of Occupational Therapy, University of Social Welfare and Rehabilitation Sciences, Tehran, Iran; ^3^ Low Vision Research Center, Department of Optometry, School of Rehabilitation Sciences, Iran university of Medical Sciences, Tehran, Iran; ^4^ Behavioral Sciences Research Center, Life Style Institute, Nursing Faculty, Baqiyatallah University of Medical Sciences, Tehran, Iran

**Keywords:** visually impaired children, occupational therapy practice framework (OTPF), functional vision tool, occupational therapy, pediatric

## Abstract

**Objective:**

Appropriate functional vision is vital for the development of visually impaired (VI) children. However, the literature currently lacks a performance-based tool for assessing functional vision, unlike the existing self-reported tools. The objective of this study is to develop and conduct a clinimetric study on a Performance-Based Functional Vision Tool (PB-FVT) specifically designed for VI children aged 3–7 and 7–10 years old.

**Methods:**

This methodological study was conducted to assess the clinimetric properties of the PB-FVT. The assessment included face validity (evaluated through cognitive interviews and an Impact Score >1.5), content validity (with criteria including content validity ratio >0.63, item content validity index >0.78, scale content validity index or average >0.8, and Kappa value >0.7), criterion validity (assessed through a concurrent test using visual acuity scores), construct validity (utilizing the known group method), relative reliability (measured by the intra-class correlation coefficient), absolute reliability (determined by the standard error of measurement and minimal detectable changes), interpretability, responsiveness, sensitivity, and specificity (analyzed via ROC curve analysis).

**Results:**

The PB-FVT was developed with 32 items, divided into five components: activities of daily living, instrumental activities of daily living, education, play, and social interaction. The results indicate that the scale demonstrates suitability in terms of validity, reliability, and other measurement characteristics.

**Conclusions:**

The valid and reliable PB-FVT may accurately assess the level of functional vision during early childhood, helping to prevent negative impacts on a child's overall development. By utilizing the PB-FVT, any functional vision impairments can be identified appropriately, enabling the planning and implementation of effective rehabilitation interventions.

## Highlights

• This study developed a Performance-Based Functional Vision Tool (PB-FVT) for children with visual impairment.
• This tool developed with 32 items into five components; activities of daily living, instrumental activities of daily living, education, play, and social interaction.
• This tool supports the planning and implementation of effective rehabilitation interventions for children ages 3–10 years old.

## Introduction

The international classification of diseases (ICD −11) (1) classified visual function into six categories: near to normal vision (vision acuity ≥20/70), moderate visual impairment (20/200 ≤ vision acuity ≤20/70), severe visual impairment (20/400 ≤ vision acuity ≤ 20/200), extremely severe visual impairment (20/1,200 ≤ vision acuity ≤ 20/400), near to blind (vision acuity ≤ 20/1,200), and absolute blind (2). Visual impairment is defined as having a vision acuity below 20/70 or a visual field of 10 degrees or less (2). Even with vision impairment, individuals can still make use of the vision they have, supporting what is known as functional vision (FV). FV refers to the effective and efficient use of the remaining visual capabilities in performing daily activities. It encompasses various aspects such as visual acuity, visual field, contrast sensitivity, visual processing skills, and the ability to integrate visual information with other sensory inputs (3). Understanding how FV limitations affect an individual's ability to engage in various occupations is essential for developing effective interventions and support strategies (4). Early vision impairment can have a detrimental impact on a child's quality of life and their FV (5). The early detection of any FV impairments is closely linked to early rehabilitation and overall well-being (6).

By 2050, there will be 360 million people with mild visual impairments and 474 million people with moderate to severe visual impairments worldwide (7). In 2020, 5.57% of the Iranian population experienced vision impairment (8). According to a study, the prevalence of visual impairment, defined as a visual acuity of 6/18, was estimated to be 0.341%, with a confidence interval of 0.187–0.571. About 1.34% of children had visual impairment in at least one eye, using the same visual acuity criterion. Specifically, 1.6% of children had visual impairment in their worse eye with a visual acuity of 6/12 or worse, while 0.24% had visual impairment in their better eye (9).

Occupational therapists must identify any deficiency in the FV of Visually Impaired (VI) children using a valid and reliable tool before implementing effective interventions. As the Table 1 shows, several FV tools have been developed for VI children.

**Table 1:** Functional vision tools for visually impaired children.

Tool	Type	Aimed age groups	Response method	Country	Gaps
Children's Visual Function Questionnaire (CVFQ) (10)	Proxy based (parents)	Above 7 years old children	Responded by parents	United States	Not performance based
First version of LV Prasad-Functional Vision Questionnaire (LVP-FVQ) (11)	Self-reported	Above 7 years old children	Responded by VI children (or proxy if needed)	India	Not performance based
LVP-FVQ II (12)	Self-reported	Around 3–4 years and around 8–13 years	Responded by VI children (or proxy if needed)	India	Not performance based
Impact of Visual Impairment on Children (IVI-C) (13)	Self-reported	Above 7 years old children	Responded by VI children (or proxy if needed)	United Kingdom	Not performance based, lack of clinimetric properties and scoring method
The Cardiff Visual Ability Questionnaire for Children (CVAQC) (14)	Self-reported	above 7 years old children	Responded by VI children (or proxy if needed)	United Kingdom	Not performance based

As Table 1 shows, the assessment of FV have involved various response methods, including child self-report to clinicians, child self-report to parents who relay the information, and parent report based on their own observations. However, each method has limitations, necessitating clinician observation. Self-reported FV measures in children may lead to overestimation or underestimation of difficulties (11). Further, clinicians rely less on parent-completed questionnaires for assessing children's issues due to communication challenges (14). As a result, technicians and professionals should not solely rely on self-reported responses when assessing functional vision in VI children (11). Instead, directly observing a child's response, whether conducted by a practitioner or researcher, is often considered a more effective approach. Furthermore, due to the evolving nature of children's activities and abilities as they grow, developing a single tool that thoroughly evaluates all aspects of their functional issues poses a significant challenge (15).

To address the aforementioned gaps, the objective of this study was to develop a valid and reliable Performance-Based Functional Vision Tool (PB-FVT) specifically designed for VI children. In the development process, the occupational part of the Occupational Therapy Practice Framework 4th Edition (OTPF-4) was utilized as the primary framework. By incorporating this framework, the researchers aimed to ensure that the PB-FVT aligns with the principles and goals of occupational therapy practice, ultimately enhancing its relevance and applicability in assessing FV in VI children (16). The occupational domain of OTPF-4 includes several categories of occupation such as activities of daily living (ADL), instrumental activities of daily living (IADL), health management, rest and sleep, work, education, play, leisure, and social participation. As we suggested that FV tasks may be related to one of the previously mentioned categories of occupation in the occupational domain of OTPF-4, it was considered as a study framework for item generation. However, other domains of OTPF-4, such as environmental factors (distance to objects), or personal factors (age, experience, skill, etc.), are also considered in the performance of FV tasks (17).

## Methods

### Study design

This study is part of a larger research project titled “Designing a FV Tool for Visually Impaired Children: A Sequential, Mixed-Methods Study,” which was conducted in two phases. The first phase involved the development of a comprehensive list of vision-related tasks in visually impaired (VI) children, and the findings from this phase were published earlier by Fard et al. (17). The ongoing second phase of the study involves the clinimetric assessment of the original 41-item PB-FVT (Supplementary Table S1). This phase involves evaluating the validity and reliability of the tool to ensure its effectiveness in assessing FV in VI children.

### Item generation

The item generation process involved two steps: a literature review and a method of directed content analysis, with the OTPF-4 serving as the underlying framework. The details of this particular aspect of the study, along with its findings, were previously described in our earlier publication (17).

### Clinimetric assessment of the tool and item reduction

#### Face validity

Both qualitative and quantitative analyses were performed to assess the face validity of the PB-FVT. In the qualitative stage, the primary version of the PB-FVT was evaluated through cognitive interviews with 10 occupational therapists who had a minimum of two years of experience working with visually impaired (VI) children. During these interviews, the therapists were requested to review the tool items and identify any items that were ambiguous or difficult to comprehend. For the quantitative assessment of face validity, another group of occupational therapists with same experience was asked to rank the significance of each item. This ranking was determined by multiplying the frequency of each item's occurrence with its perceived importance (18).

#### Content validity

To ensure content validity, a qualitative assessment was conducted by soliciting feedback from 10 experts in the field of occupational therapy. These experts, ranging from assistant professors to full professors, possessed diverse credentials, expertise, and years of practice, with experience ranging from 4 to 25 years in occupational therapy and rehabilitation. They were recruited from various practice settings, including both academic institutions and clinical settings.

The selected experts were specifically requested to provide their expert opinions on multiple aspects of the PB-FVT, including the appropriateness of language, grammar, terminology, and item placement. Their valuable feedback and suggestions were carefully considered in order to enhance the content validity of the questionnaire.

The content validity ratio (CVR) was determined using Lawshe's approach. Items that scored 0.62 or above, according to Lawshe's table, were retained (19). Lawshe's table is a reference table used in content validity analysis. It provides guidelines for determining CVR based on the number of experts participating in the evaluation. The table helps researchers determine the minimum acceptable level of agreement among experts for each item being evaluated (19). To check CVR, another group of 10 experts, possessing the same characteristics as the previous group, were asked to rate each of the 41 items of the PB-FVT as either “essential,” “helpful but not essential,” or “unnecessary.” Additionally, the content validity index (CVI) was assessed by obtaining the perspectives of ten new experts who possessed the same characteristics as the previous group. These experts assessed the relevance of items using a 4-point Likert scale, ranging from “not relevant” to “completely relevant.” Subsequently, the item-level content validity index (I-CVI), Kappa statistic, and scale-level content validity index average (S-CVI/Ave) were calculated (20). An excellent level of agreement is indicated by a Kappa value greater than 0.74, while S-CVI/Ave score of 0.9 or higher suggests a highly favorable content validity (21).

### Criterion and construct validities

The criterion and construct validities of the tool were evaluated using a sample of 122 visually impaired (VI) children. The recruitment process utilized a census sampling method, ensuring the inclusion of all eligible visually impaired (VI) children. This approach involved inviting every VI child between the ages of 3 and 10 to participate in the study. The assessment was conducted by two occupational therapists with master's degrees in four schools for visually impaired children and one children's rehabilitation center in a large city in Iran, between March and June 2022. To ensure ethical considerations, consent forms were obtained from parents and authorities.

The inclusion criteria for participant selection were as follows: the children had to be between the ages of 3 and 10 years old, have varying levels of vision impairments excluding only those with light perception or absolute blindness, as determined by visual acuity scores obtained from their medical records. The eligible participants were either attending specialized schools for visually impaired children or had been referred to occupational therapy and rehabilitation centers. Exclusion criteria were applied to children who were unable to perform all the tasks related to the tool.

For the criterion validity assessment, a concurrent test method was employed, using visual acuity scores as the reference standard. Initially, the visual acuity scores of the children were obtained from their medical records. The samples were then categorized into two groups: the first group consisted of visually impaired (VI) children with mild to moderate impairment, while the second group comprised those with severe impairment to near blindness. The correlation between the visual acuity scores and FV scores was calculated using Spearman's test.

In terms of construct validity, the same sample used for the criterion validity stage was utilized. The construct validity was evaluated using the known groups' method. The researchers hypothesized that the FV scores of the group aged 7–10 years would be higher than those of the group aged 3–7 years. Due to the non-normal distribution of the data, the Mann-Whitney test was employed to compare the FV scores between the two age groups, enabling an analysis of whether the scores differed significantly based on the age categories.

### Reliability

To evaluate the reliability of the PB-FVT, both relative and absolute reliability measures were employed. Relative reliability was assessed using the interclass correlation coefficient (ICC) with the inter-rater method. Two occupational therapists independently observed the FV of 50 VI children and completed the PB-FVT twice, with a two-week interval between assessments. To assess the absolute reliability of the PB-FVT, the Standard Error of Measurement (SEM) and Minimal Detectable Changes (MDC) were calculated. The SEM represents the measurement error associated with the tool, while the MDC reflects the minimum amount of change in FV scores that can be considered beyond measurement error (22).

The Standard Error of Measurement (SEM) and Minimal Detectable Changes (MDC) were calculated using the following formula: SEM = SD Pooled √1– ICC (21), and MDC = SEM X 1.96 X √2 and MDC% = (MDC /mean) * 100, where mean is the mean for all the observations from test sessions 1 and 2. A MDC percentage of 30% is considered acceptable, whereas values under 10% are considered excellent (23).

### Interpretability

Further, to analyze interpretability, the percentage of unanswered items and ceiling and floor effects were reported. The sample from the construct validity assessment stage (*n* = 122) was used to assess the percentage of unanswered items, ceiling and floor effects. The optimal values for the percentage of unanswered items and the ceiling and floor effects are 10%–20% (24) and <15% (25), respectively. Furthermore, to assess interpretability, the minimal important change (MIC) was determined using the following formula: MIC = 0.5 * SD of Δ score.

### Sensitivity, specificity, cutoff point, and scoring

Based on their visual acuity scores, VI children were divided into two groups: those with mild to moderate visual acuity and those with severe to near-blind visual acuity. The receiver operating characteristic (ROC) was plotted to determine the appropriate sensitivity, characteristic, and cutoff point using the data of 122 VI children in GraphPad Prism 9 software. There are five areas under the ROC curve: excellent = 90–100, good = 80–90, relatively good = 70–80, weak = 60–70, and useless = 50–60 (26). As a rule, if a test is capable of detecting accurately, the ROC curve above the square diameter will approach the ideal state of area 1 (26).

### Translation

The final draft of PB-FVT underwent a translation process from Persian to English by native Persian speakers who also possessed proficiency in English. This translation process entailed multiple stages, including forward translation, back-translation, and review by bilingual experts. These measures were taken to ensure the accuracy and equivalence of meaning between the original language and the target language, English.

### Ethical considerations

The University of Social and Welfare Rehabilitation Science (USWR) Ethics Committee gave its approval to this study (IR. SBMU.PHARMACY.REC.1399.218). The study's participation was entirely voluntary and subject to consent. The PB-FVT was filled out anonymously without an identification number in order to protect the confidentiality of the participants' data.

## Results

### The primary PB-FVT

Initially, a total of 496 tasks related to FV were discovered through a combination of literature review (based on 23 articles) and interviews with participants (involving 16 participants) (17). After eliminating duplicate tasks, the number was reduced to 143. Through subsequent merging of similar tasks, the final selection consisted of 41 distinct tasks. Therefore, a primary 41-item PB-FVT was incorporated into the clinimetric assessment stage.

### Clinimetric assessment stage

In the present study, a total of 122 visually impaired (VI) children participated, with 43% (52) in the 3–7 age group and 57% (70) in the 7–10 age group. The gender distribution was relatively balanced, with 49.2% (60) male and 50.8% (62) female participants. Regarding visual acuity, 45.9% (56) of the participants had vision acuity of ≥20/70, 54.1% (66) had vision acuity ranging from 20/400 to 20/200, and smaller percentages fell into the other two acuity groups. In terms of settings, the majority of participants attended schools, with School C having the highest attendance rate of 25.4% (31), followed by School D with 30.3% (37) of the participants. Additionally, 12.3% (15) of the participants received rehabilitation services at a specialized center (Table 2).

**Table 2:** The demographic characteristics of VI children.

Groups			3–7 aged group		7.1–10 aged group		Total	
Variables			*N*	%	*N*	%	*N*	%
Age groups			52	43	70	57	122	100
Gender	Male		25	48.08	35	50	60	49.2
	Female		27	51.92	35	50	62	50.8
Visual acuity	Group 1	Vision acuity ≥20/70	8	6	17	14	56	45.9
		20/200 ≤ vision acuity ≤20/70	11	9	20	16		
	Group 2	20/400 ≤ vision acuity ≤20/200	10	8	8	6	66	54.1
		20/1,200 ≤ vision acuity ≤20/400	18	12	20	16		
		vision acuity ≤20/1,200	5	4	6	4		
Settings	Schools	School A	6	11.54	11	9	17	13
		School B	6	11.54	16	13	22	18
		School C	12	23.7	19	27.14	31	25.4
		School D	13	25	24	34.29	37	30.3
	Rehabilitation center	A specialized center	15	28.85	0	0	15	12.3

### Face validity

In terms of qualitative face validity, the primary 41-item PB-FVT resulted in 10 items being modified based on their clarity, relevance, and comprehensibility (items of 2, 4, 8, 9, 11, 17, 18, 21, 26, and 29). Then, the rechecking process was carried out with the same occupational therapist to ensure that the revised items aligned with the desired criteria for face validity. In terms of quantitative face validity, all items were scored above 1.5 up to 4.9, except item 8 for the 3–7 age group. This item was scored 0.28, therefore it was revised. The 41-item PB-FVT was entered into the next step of the process.

### Content validity

During the content validity assessment, expert recommendations were utilized to refine the items. Out of the original 41 items, 9 items (15, 16, 18, 19, 20, 21, 23, 34, 39) were removed based on their respective CVR scores of 0.2, 0, 0.3, 0, 0.4, 0, 0.2, 0.2, and 0. These items included tasks such as cleaning eyeglasses with a cloth, clipping the index finger nails from each hand while sitting at a table and throwing them into the trash (specifically for the 7–10 age group), dropping a ball into a basket after passing an obstacle, finding four different types of balls (basketball, goal ball, soccer ball, and small baseball ball) arranged in a specific pattern within a room, walking on a straight line measuring one meter for children aged 3–6, heel-toe walking on the same line for children aged 7–10, filling a sprinkler from a faucet and watering a pot, dialing the number 0912835764 on a phone using the sense of sight, and playing a specific game with karts, as well as recognizing 2, 5, 10, and 50 thousand tomans banknotes.

The CVI assessment indicated that all remaining items had an I-CVR above 0.8 and a modified Kappa coefficient above 0.79, demonstrating strong content validity. Furthermore, the S-CVI/Ave scores for the age groups 3–7 and 7–10 were 0.88 and 0.89, respectively,

### Criterion validity

Spearman's test revealed a significant negative correlation (*P* < 0.001) between visual acuity and FV scores across all components of the tool as well as the total score. This association was observed in both age groups, with a sample size of 52 in the 3–7 age group and 70 in the 7–10 age group (Table 3).

**Table 3:** The correlation between FV and visual acuity scores of VI children.

Components	Age group^a^	Correlation rate	*P*-value^b^
ADL	1	−0.60	*P* < 0.001
	2	−0.68	*P* < 0.001
IADL	1	−0.60	*P* < 0.001
	2	−0.67	*P* < 0.001
Education	1	−0.65	*P* < 0.001
	2	−0.87	*P* < 0.001
Play	1	−0.61	*P* < 0.001
	2	−0.89	*P* < 0.001
Social interaction	1	−0.71	*P* < 0.001
	2	−0.93	*P* < 0.001
Total score	1	−0.68	*P* < 0.001
	2	−0.90	*P* < 0.001

^a^ Group 1 = 3–7, group 2 = 7–10.

^b^ Spearman's test.

### Construct validity

The findings of the Mann-Whitney test indicated a significant differentiation in the FV scores between the two age groups (*P*-value 0.05). Table 4 provides a comprehensive comparison of the FV scores between the age groups of 3–7 years and 7–10 years. These findings support the construct validity of the tool, suggesting that it effectively discriminates between different age groups of VI children based on their FV scores.

**Table 4:** Comparison of the FV score in the first (3–7 years) and the second (7–10 years) age groups.

Components	Age group^a^	Mean score	Test statistics	*P*-value
ADL	1	47.37	3.83	*P* < 0.001
	2	72.00		
IADL	1	47.57	3.96	*P* < 0.001
	2	71.85		
Education	1 s	38.11	6.41	*P* < 0.001
	2	78.88		
Play	1	50.49	3.05	*P* < 0.001
	2	69.68		
Social interaction	1	48.70	3.48	*P* < 0.001
	2	71.01		
Total score	1	44.13	4.67	*P* < 0.001
	2	74.40		

^a^ Group 1 = 3–7, group 2 = 7–10.

### Reliability

Table 5 demonstrates excellent relative and absolute reliabilities of the PB-FVT (across various components. The ICC for ADL, IADL, Education, Play, Social interaction, and Total score ranged from 0.978 to 0.995, with corresponding confidence intervals indicating high reliability. Additionally, the SEM and MDC values were consistently low, further supporting the excellent reliability of the PB-FVT in assessing functional vision in visually impaired children.

**Table 5:** Excellent relative and absolute reliabilities of the PB-FVT in VI children.

Components	Score range	ICC	CI 95%	*P*-value	SEM	MDC	MDC%	Interpretation
ADL	15–75	0.978	0.751–993	*P* < 0.001	2.95	8.17	6.05	Excellent
IADL	1–5	0.980	0.970–0.986	*P* < 0.001	0.18	0.49	5.21	Excellent
Education	7–35	0.995	0.992–0.997	*P* < 0.001	1.09	2.99	4.3	Excellent
Play	3–15	0.995	0.993–0.997	*P*-Value	0.36	0.99	3.21	Excellent
Social interaction	6–30	0.990	0.933–0.996	*P* < 0.001	0.82	2.27	6.89	Excellent
Total score	32–160	0.993	0.792–0.998	*P* < 0.001	3.34	9.17	7.64	Excellent

### Interpretability

The PB-FVT demonstrated minimal ceiling and floor effects, with only 4% of participants achieving the maximum score and none achieving the minimum score for the entire tool. The subscales also showed low ceiling and floor effects, ranging from 0.05 to 0.12. The absence of missing items further indicates the completeness of the data. Additionally, the MIC scores ranged from 0.16 to 1.05, further supporting the interpretability of the PB-FVT in assessing functional vision in visually impaired children.

### Sensitivity, specificity, and cut off score

The AUC of the PB-FVT (Supplementary Figure S1), for the age groups of 3–7, 7.1 −19 years old were 0.91 (CI 95% = 0.83–0.99, *P* < 0.001) and 0.97 (CI 95% = 0.94–1, *P* < 0.001), respectively. The PB-FVT had a cutoff score of 91 for the age group of 3–7, achieving a sensitivity of 96.88% and specificity of 78.95%. Similarly, for the second age group, the cutoff score was 104, with a sensitivity of 97.06% and specificity of 86.18%. These findings indicate that the PB-FVT has good discriminative ability in identifying functional vision impairment in different age groups, with high sensitivity in correctly identifying children with impaired functional vision and relatively high specificity in correctly identifying children without impairment.

### Final version of the PB-FVT

The final version of the 32-item PB-FVT is shown in Supplementary Table S2. The first five items have separate questions for the two age groups, while items 21 and 23 are explicitly for the 7.1–10 age group. The remaining items are applicable to both age groups. The ADL component (Items 1–16) of the PB-FVT involved tasks such as dressing and undressing, pouring water from a pitcher, peeling fruit, eating, brushing teeth, and using the toilet. IADL was evaluated through item 16, which specifically addressed table setting. The education component (Items 17–23) of the PB-FVT included tasks related to painting, writing, reading, and using a mobile phone. The play component (Items 24–26) comprised a sensory-motor game, a symbolic game, and a constructive game. Lastly, social interaction was assessed through items 27–32, which focused on tasks involving facial recognition, communication skills, and social mobility.

This tool must be administered by an occupational therapist as observers to measure the FV of visually impaired children. Each item of the PB-FVT is scored based on a Likert scale ranging from 1 to 5, where a score of 1 indicates an inability to perform a function, whereas a score of 5 suggests correct performance. Better FV results in higher scores, and vice versa. As mentioned, the cutoff score of the PB-FVT for the age groups of 3–7 and 7–10 was 91 and 104, respectively.

## Discussion

Our research highlights the absence of a specifically developed performance-based FV assessment tool for VI children. The newly developed PB-FVT offers the advantage of being performance-based, allowing VI children to complete tasks while being observed by clinical practitioners or researchers. This approach was not previously utilized in FV tools due to challenges in replicating real-world settings such as classrooms, including factors like lighting levels and sitting distance. To address these issues, tasks were identified that could be conducted in clinical settings, making the current tool more applicable for use by clinical practitioners, eye care professionals, and researchers.

Assessing FV in children can pose challenges due to the evolving nature of their activities as they grow older, making it difficult to create a single comprehensive tool for evaluation (11). However, an additional challenge lies in selecting the appropriate items for assessment. It is crucial to ensure a balanced distribution of difficulties that aligns with the abilities of the patients, as unsatisfactory results may arise if this balance is not achieved (11). In this study, specific items were designed separately for the age groups of 3–7 and 7–10, aiming to address these challenges and enhance the tool's suitability for evaluating FV in different age groups.

The 32-item PB-FVT consisted of five components: ADL, IADL, play, education, and social interaction. he ADL component of the PB-FVT excludes items related to sexual activity and personal device maintenance, as these tasks are typically applicable to adult populations, as outlined by the OTPF-4 (27). Similarly, the IADL component of our tool focuses on meal preparation and clean-up, which are activities that can be performed by children, while recognizing that other IADL tasks such as driving, community mobility, and financial management are typically limited to adults according to the OTPF-4 (27). Also, the tasks within the education component were intentionally designed to facilitate easy implementation in clinical settings. Furthermore, the items developed for the play and social interaction components were specifically tailored to match the abilities and developmental level of children.

Regarding clinimetric assessment, the primary 41-item tool had optimal face validity, however, during quantitative content validity, 9 items were removed. The 32 remained items indicate had optimal I-CVI, S-CVI, and Kappa coefficient of the agreement.

For criterion and construct validity, we recruited all available VI children to ensure a diverse sample. We invited participants from schools for VI children and clinical settings such as occupational therapy centers to participate in the study. The sample encompassed a range of visual acuity scores, spanning from near-normal vision (visual acuity of 20/70) to near-blindness with only light perception or complete blindness. To facilitate analysis, we divided the participants into two groups based on their visual acuity (28). Our developed tool further demonstrated its construct validity by yielding significantly different FV scores for the two groups of participants. This finding supports the notion that the tool effectively captures and differentiates the FV abilities of VI children across varying levels of visual acuity (29). Regarding the exploratory factor analysis, we did not employ this technique as it is not suitable for analyzing “formative” scales. The difference between “formative” scales and “reflective” scales lies in the relationship between the observed variables and the underlying construct they are intended to measure. Exploratory Factor Analysis (EFA) and Confirmatory Factor Analysis (CFA) are typically applied to measure “reflective” scales, where items are considered to be indicators of underlying latent constructs. In reflective measurement models, the latent construct is assumed to cause the observed variables or items. In the case of our tool, the items were designed to assess specific functional tasks rather than serving as indicators of an underlying construct. Hence, an exploratory factor analysis was not deemed appropriate for our study.

The 19-item LVP-FVQ, utilizing a 5-point Likert scale, was developed for VI children between the ages of 8 and 18. Its purpose was to assess general visual functioning, encompassing tasks such as lacing shoes, walking in school corridors, and threading a needle. However, the LVP-FVQ has encountered psychometric challenges, including inadequate measurement accuracy and a lack of comprehensive dimensionality assessment. Furthermore, there is a dearth of items addressing mobility and technology-related aspects. In comparison, the revised 23-item LVP-FVQ II demonstrates significant improvements in terms of enhanced psychometric properties when compared to the original version (12). The PB-FVT stands out in comparison to the LVP-FVQ due to its robust clinimetric assessment. While the item generation process for the LVP-FVQ relied on a literature review and interviews, it lacked a primary conceptual framework. In contrast, the present study for the PB-FVT employed the OTPF as the guiding framework. This utilization of the OTPF-4 provided a solid foundation and conceptual framework for the development and implementation of the PB-FVT, enhancing its validity and relevance to occupational therapy practice.

The 25-item CVAQC measures the difficulty of doing tasks with regard to education, near vision-related tasks, distance vision-related tasks, getting around, social interaction, entertainment, and sports in VI children aged 5–15 years (14). The validity and reliability of the tool were assured using Rash analysis, content validity, construct validity, and temporal stability. However, it is important to note that the item generation process for the CVAQC differed from the present study. In the CVAQC, items were generated through a focus group involving children and young people. In contrast, the current study relied on a literature review and interviews to generate the items for the assessment tool.

When children are unable to offer self-report data, the CVFQ scale, which was created for children up to the age of 7, can be helpful (12). The CVFQ is a parent-proxy report scale, unlike the LVP-FVQ, which is self-reported. Therapeutic professionals, however, typically solely rely on proxy responses. The PB-FVT, on the other hand, was developed to assess a rehabilitative or occupational therapist's perception of a VI child's capacity for carrying out vision-specific tasks such as working with a phone and reading menus. The current FV tool is performance-based and, to some extent, age-specific, which can boost its validity more than other tools.

### Implications for occupational therapy practice

The development of the PB-FVT holds significant implications for occupational therapy practice, offering a reliable and valid tool specifically designed to assess the functional capacity of VI children. By utilizing the PB-FVT, occupational therapists can gain valuable insights into the abilities and challenges of VI children in various functional domains. One notable advantage of the PB-FVT is its ease of use, making it accessible for occupational therapists to administer during assessments. The tool incorporates common household items, eliminating the need for specialized equipment and resources. This practicality enhances the feasibility of incorporating the PB-FVT into routine clinical practice, allowing for efficient assessment of functional vision in VI children. Compared to existing functional vision tools, the PB-FVT may offer improved reliability and accuracy. By employing a performance-based approach, the PB-FVT enables VI children to actively engage in tasks while being observed by occupational therapists. This direct observation allows for a more comprehensive and objective evaluation of their FV. Furthermore, the PB-FVT takes into account the unique developmental needs of VI children across different age groups. By tailoring specific items to the age ranges of 3–7 and 7–10, the tool ensures that the assessment tasks are developmentally appropriate and align with the functional expectations for each age group.

### Limitation

Due to the formative nature of the PB-FVT, traditional methods such as EFA, CFA, and Cronbach's alpha were not suitable for assessing its validity and reliability. This posed a limitation in our study. Additionally, the accessibility of VI children was another constraint. We focused on those who were referred to occupational therapy centers for rehabilitation or attended schools specifically catering to VI children. However, it is important to note that we included all available samples using the census sampling method. The PB-FVT scale was originally developed in Persian and subsequently translated into English. It is important to acknowledge that cultural differences can potentially affect the interpretation and understanding of the scale items, which in turn may affect its validity and reliability when used in English-speaking countries. However, it is worth noting that during the item generation phase, international literature was consulted, and the final selection of items demonstrated general alignment. Additionally, the translation process from Persian to English was conducted meticulously, involving both forward and back-translation by accomplished bilingual experts. All items were duly adjusted to ensure the utmost accuracy and equivalence. As a result, it can be argued that the PB-FVT scale holds potential suitability for use in English-speaking countries. However, it is essential to conduct further psychometric assessments and validation studies to establish its validity and reliability in the specific context of the target population.

## Conclusion

The PB-FVT is a reliable, valid and safe tool to assess FV proficiency in VI children. Due to the tool's usage of the OTPF-4, which is comprehensive and multidimensional, it may serve as a representative of the activities required for children with ages of 3–7 and 7.1–10. As a result, it can be used by practitioners and researchers to measure the level of FV in VI children in their early years. Therefore, effective rehabilitation can be planned before visual impairment negatively affects a child's quality of life and general development.

## Data Availability

The datasets presented in this study can be found in online repositories. The names of the repository/repositories and accession number(s) can be found in the article/**Supplementary Material**.
