# Supplementary material for: Development and clinimetric assessment of a performance-based functional vision tool in visually impaired children
Source: Front Pediatr. 2023 Nov 1;11:1275726. doi: 10.3389/fped.2023.1275726 (PMC10656732; doi:10.3389/fped.2023.1275726)
Supplement: Supplementary file 1 [file Datasheet1.docx]

Development and clinimetric assessment of a performance-based functional vision tool in visually impaired children

Fatemeh Ghasemi Fard^[[1]](#footnote-1)^, Hooshang Mirzaie*^[[2]](#footnote-2)^, Seyed Ali Hosseini^[[3]](#footnote-3)^, Abbas Riazi^[[4]](#footnote-4)^, Abbas Ebadi^[[5]](#footnote-5)^, Narges Hooshmand Zadeh^[[6]](#footnote-6)^.

**Running title:**

Vision-related tasks in Visually Impaired Children

**Corresponding author:**

Hooshang Mirzaie, Department of Occupational Therapy, University of Social Welfare and Rehabilitation Sciences, Tehran. Iran, Tel: +989122208426, Email: fg.star1373@gmail.com

1. . A: Pediatric Neurorehabilitation Research Center, University of Social Welfare and Rehabilitation Sciences, Tehran, Iran.

   B: Department of Occupational Therapy, University of Social Welfare and Rehabilitation Sciences, Tehran. Iran. [↑](#footnote-ref-1)
2. . A: Pediatric Neurorehabilitation Research Center, University of Social Welfare and Rehabilitation Sciences, Tehran, Iran.

   B: Department of Occupational Therapy, University of Social Welfare and Rehabilitation Sciences, Tehran. Iran. (Corresponding author). [↑](#footnote-ref-2)
3. . Department of Occupational Therapy, University of Social Welfare and Rehabilitation Sciences, Tehran. Iran. [↑](#footnote-ref-3)
4. . Low vision Research Center, Department of Optometry, School of Rehabilitation Sciences, Iran university of Medical Sciences, Tehran, Iran. [↑](#footnote-ref-4)
5. . Behavioral Sciences Research Center, Life style institute, Nursing Faculty, Baqiyatallah University of Medical Sciences, Tehran, IR Iran. [↑](#footnote-ref-5)
6. . Department of Occupational Therapy, University of Social Welfare and Rehabilitation Sciences, Tehran. Iran.

   *Corresponding author: Hooshang Mirzaie, fg.star1373@gmail.com [↑](#footnote-ref-6)
